# A View on Molecular Complexity from the GDB Chemical Space

**DOI:** 10.1021/acs.jcim.5c00334

**Published:** 2025-05-15

**Authors:** Ye Buehler, Jean-Louis Reymond

**Affiliations:** † Department of Chemistry, Biochemistry and Pharmaceutical Sciences, 27210 University of Bern, Freiestrasse 3, 3012 Bern, Switzerland

## Abstract

One recurring question when choosing which molecules to select for investigation is that of molecular complexity: is there a price to pay for complexity in terms of synthesis difficulty, and does complexity have anything to do with biological properties? In the chemical space of small organic molecules enumerated from mathematical graphs in the GDBs (Generated DataBases), most compounds are too complex and challenging for synthesis despite containing only standard functional groups and ring types. For these GDB molecules, we find that an increasing fraction (MC1) or number (MC2) of non-divalent nodes in the molecular graph represent simple measures of molecular complexity, which we interpret in terms of potential synthesis difficulties. We also show that MC1 and MC2 are applicable to commercial screening compounds (ZINC), bioactive molecules (ChEMBL) and natural products (COCONUT) and compare them with previously reported measures of molecular complexity and synthetic accessibility.

## Graphical abstract

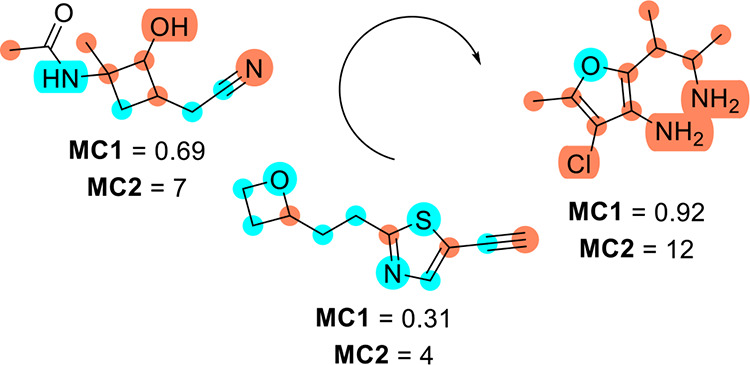

In medicinal chemistry, chemical space describes the ensemble of all molecules of interest for drug discovery,^1,2^ usually small molecules within the Lipinski Ro5 limits,^3^ but also larger modalities^4^ such as peptides,^5^ macrocycles^6^ and bifunctional chimera.^7^ All these molecules add up to big data sets, for example, the open access collections ZINC (commercial screening compounds),^8^ ChEMBL (reported molecules and their bioactivity)^9^ or COCONUT (natural products),^10^ not to mention ultralarge combinatorial spaces containing molecules made of connected building blocks.^11,12^

One recurring question when choosing which molecules to select for investigation is that of molecular complexity: is there a price to pay for complexity in terms of synthesis difficulty, and does complexity have anything to do with biological properties? Although not unequivocally defined, molecular complexity represents the diversity and intricacy of substructures in a molecule and is indeed meant to indicate how difficult it might be to assemble this molecule from simple components.^13–15^ For the synthetic organic chemist there is an irresistible fascination with structurally complex molecules and more specifically natural products because they are obviously possible since nature made them, but they often offer unprecedented challenges, providing a key driving force for developing new synthetic methods.^16,17^ However, synthetic efficiency also means to aim for the simplest possible synthetic route and therefore striving for lower complexity.^18,19^ Medicinal chemists are also fascinated by complexity in a similar love-hate relationship: structurally simple molecules are elegant and desirable and might even have a higher chance of showing target binding in an initial phase of a project when fragments are being screened.^20–22^ On the other hand, molecules tend to become more complex during medicinal chemistry optimization where substituents such as methyl groups, chlorine atoms or fluorine atoms are added to increase potency, selectivity, or metabolic stability.^23–26^ Computational chemists have followed on this question by defining various measures of molecular complexity, which are computed from the molecular structure alone,^13,14,27–40^ as well as measures of synthetic accessibility, which are computed taking into account the similarity to known molecules optionally classified as hard or easy to synthesize, or the estimated number of synthetic steps from known precursors to make the molecule.^41–45^ All of these measures have in common that they are themselves complex and nontrivial to interpret and that they have been designed taking series of known molecules into account. A subset of these measures which can be computed with freely accessible tools are summarized in Table 1.

**Table 1:** Selected Molecular Complexity and Synthetic Accessibility Measures

Name	Description	Ref.
*Molecular Complexity*		
FCFP4	Number of on-bits in a binary 2048-bit FCFP4 fingerprint	^30^
Data Warrior	Determines all distinct substructures for every bond count up to seven bonds, and the maximum value is used to calculate the fractal complexity using the Minkowski–Bouligand (box-counting) dimension concept	^31^,^36^
Böttcher	Shannon entropy using additive atom contributions considering valence electrons, atom environment, chirality and molecular symmetry	^33^
Proudfoot	Shannon entropy using additive atom contributions considering atomic number, the number of connections and paths up to length 2.	^35^
SPS	Sum of heavy atom contributions considering hybridization, stereochemistry, nonaromaticity, and the number of heavy-atom neighbors	^39^
nSPS	SPS normalized to heavy atom count	^39^
*Synthesizability*		
SAscore	Presence of fragments frequently encountered in PubChem molecules combined with a complexity penalty considering ring types, stereochemistry and molecule size	^41^
SCS	Machine-learned score from 12 million reaction in Reaxys predicting the number of steps required for synthesis from common starting material from the ECFP4 fingerprint as input, with maximum value 5	^42^
*This Work*		
MC1	Fraction of non-divalent nodes in the molecular graph	
MC2	Number of non-divalent nodes not considering C=O groups in N—(C=O) and O—(C=O) substructures	

In our own adventures in chemical space, we have been confronted with complexity in the generated databases (GDBs), which enumerate billions of hypothetical molecules obtained by substituting non-hydrogen atoms (C, N, O, S and halogens) for graph nodes and single, double or triple bonds for graph edges in mathematical graphs, an approach which we have realized with graphs up to 17 nodes.^46–50^ Although we applied rules limiting ring strain and functional group diversity to only retain molecules which were in principle stable and synthetically possible, most GDB molecules combined polycyclic ring systems with multiple substituents, functional groups, and chiral centers. In short, nothing was wrong with these molecules, but everything was challenging. While we chose relatively simple cases for synthetic realization,^51–56^ we later focused our GDB-databases on subsets by applying additional criteria such as fragment-likeness,^57^ drug-likeness^58^ and ChEMBL-likeness.^59^ However, even in our latest and most restrictive subset GDB-13s, comprising 99.4 million molecules defined by keeping only molecules featuring very simple functional groups and ring systems among the 977 million molecules in the database GDB-13,^60,61^ most molecules still presented daunting challenges for synthesis.

Analyzing our restricted subset of GDB-13s molecules in view of their experimental synthesis considering our most recent syntheses of GDB molecules^62–64^ has led us to a surprisingly simple definition of molecular complexity, which is based on the fraction or number of divalent heavy (non-hydrogen) atoms (e.g., —CH_2_—, —CH=, —C≡, —O—, —NH—, =N—, —S—) in a molecule as a mark of low complexity, whereby we consider molecular complexity in a synthetic accessibility perspective, although without considering the availability of starting materials, as explained below.

Our first molecular complexity measure MC1 is the fraction of non-divalent nodes in the molecular graph and is independent of molecule size (eq 1, the molecular graph can be represented as the hydrocarbon resulting from setting all bonds to single bonds and all heavy atoms to carbon). MC1 reflects the fact that every additional branching point in a molecule (a trivalent or tetravalent node) means trouble for synthesis in the form of a new ring or substituent (often with monovalent nodes) requiring additional synthesis steps to be installed, possibly undesired chemical reactivity and the need for protecting groups (typically if the added substituent is an alcohol or amine), and sometimes the formation of a stereogenic center at the branching point resulting in multiple stereoisomers requiring separation or a stereoselective synthesis. Furthermore, steric hindrance renders many reactions inoperative at the branching point itself or at reactive sites close to the branching point. By contrast, adding new divalent nodes in the molecular graph simply extends a chain or ring size, which most often does not create the above-mentioned problems in terms of additional stereochemistry, need for protecting groups, or increased steric hindrance reducing reactivity, and can even often simply be handled by choosing an extended building block without redesigning the synthesis. Note that MC1 does not provide informative values for very small molecules. For example, trifluoroacetic acid or *tert*-butanol give the maximum possible value MC1 = 1 although they are not complex. Similarly, the MC1 values are not informative for polymers. Note for example the decreasing MC1 values (not considering cross-linkers) in the series cellulose (MC1 = 0.82), polylactic acid (MC1= 0.8), polypropylene (MC1 = 0.67), silk fibroin (MC1 = 0.64), Kevlar (0.44), polyethylene terephthalate (MC1 = 0.43), polystyrene (MC1 = 0.25), nylon-6 (MC1 = 0.25), polyethylene (MC1 = 0) and polyethylene glycol (MC1 = 0).

Our second molecular complexity measure MC2 takes the same view but considers that complexity increases with molecule size, measured as the heavy atom count (HAC), but only if branching increases. Therefore, divalent atoms are not counted. Furthermore, we also do not count carboxyl groups in acids, esters, amides, carbonate, carbamates, or ureas because their synthesis is very often straightforward (eq 2). We however consider atoms in functional groups such as amidines, guanidines, thioesters, thiones, sulfoxides, sulfinates, sulfones, and sulfonamides, as well as in phosphorus containing functional groups for calculating MC2 because their chemistry is not as versatile as for the above-mentioned carboxyl derivatives. By this second measure, small molecules are often simple. On the other hand, large and linear molecules such as fatty acids and their derivatives remain simple, while complex polycyclic and substituted molecules are complex. Due to its size dependence, MC2 cannot give useful values for polymers.

1 MC1=1−FDV

FDV = fraction of divalent nodes in the molecular graph.

MC2=NDV 2

NDV = number of non-divalent nodes in the molecular graph, not counting C=O in (X—C=O) for X = N or O.

To illustrate how our complexity measures work, we have assembled four TMAPs (tree-maps),^65^ organized by substructure similarity using the MAP4C molecular fingerprint,^66^ each consisting of 30,000 randomly selected molecules from the databases GDB-13s,^61^ ZINC,^8^ ChEMBL^9^ and COCONUT.^10^ Each molecule is a point on the TMAP and is color-coded according to MC2, with a low, middle, and high complexity example featured for each data set (Figure 1).

**Figure 1:**
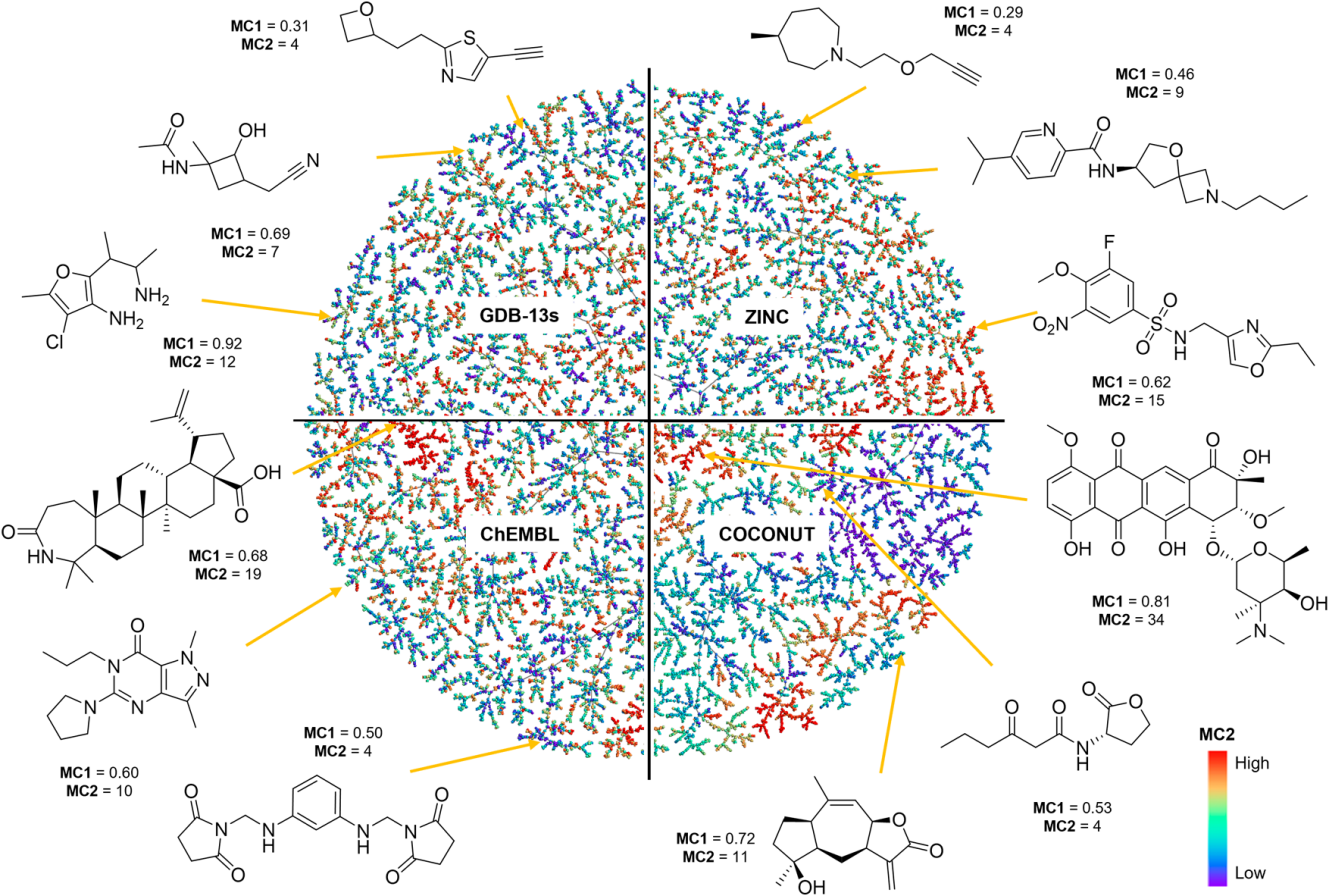
Partial views of the TMAP visualization of random subsets of 30,000 molecules each from the databases GDB-13s (upper left, full interactive TMAP available at https://tm.gdb.tools/MAP4C/GDB-13s_complexity), ZINC (upper right, full interactive TMAP at https://tm.gdb.tools/MAP4C/ZINC_complexity), ChEMBL (lower left, full interactive TMAP available at https://tm.gdb.tools/MAP4C/ChEMBL_complexity) and COCONUT (bottom right, full interactive TMAP available at https://tm.gdb.tools/MAP4C/COCONUT_complexity) assembled using the MAP4C molecular fingerprint to compute similarity between molecules and color-coded by MC2 values from purple (lowest value) to red (highest value), with examples of high, middle, and low molecular complexity molecules.

In the full size interactive versions of these maps,^67^ color-codes are also available for MC1 and for the following molecular complexity and synthetic accessibility scores for which the code was available (see also Table 1): a) FCFP4, the bit count of a 2048-bit fingerprint normalized to HAC;^30^ b) Data Warrior complexity score based on fractal complexity;^31^ c) Böttcher’s C_m_, an entropy-based additive complexity score considering stereochemistry and atomic environments;^33^ d) Proudfoot’s C_M_, a path based complexity score based on graph theory;^35^ e) SPS and nSPS, spacial score and normalized spacial score;^39^ f) SAscore, a synthetic accessibility score based on the occurrence of known fragments combined with overall complexity;^41^ g) SCS, a machine-learned synthetic complexity score based on Reaxys reaction data;^42^ h) HAC, a measure of molecule size. These TMAPs illustrate which molecules fall within the low versus high complexity range of each measure.

One key difference between MC1/MC2 and most other molecular complexity measures is that we do not distinguish between sp^2^ and sp^3^ branching points since we do not count the fraction of sp^3^ atoms and/or the number of chiral centers, although these often indicate complexity and have been linked to better drug properties.^68,69^ Our choice is motivated by the presence of unusual substitution patterns in aromatic or heteroaromatic rings in GDB molecules which would be difficult to prepare and the fact that the functionalization of aromatic and heteroaromatic rings^70^ and the control of atropisomerism in biaryls^71^ are both challenging. Note that setting all branching points as contributing to complexity implies that carbohydrates and polyphenols, which are often considered simple because they occur in abundant and inexpensive biomass,^72^ receive a high complexity score.

Further insights are provided by analyzing the correlation between pairs of molecular complexity and synthetic accessibility scores. For the case of GDB-13s for which almost all molecules have the same size (HAC = 13), the different complexity measures do not correlate with each other (*r* ^2^ < 0.6), except for MC1 with MC2 and SPS with nSPS, which is not surprising since MC1 is approximately the size-independent version of MC2, and the same applies to nSPS and SPS (note that nSPS and SPS also correlate with the synthetic accessibility score SAscore). In the case of COCONUT, ZINC and ChEMBL which span a broad range of molecular sizes, several of the complexity measures correlate with HAC, and therefore with each other (Figure 2a). The sensitivity of each measure to molecule size as measured by HAC is also well visible in the frequency histograms according to each measure and each data set (Figure 2b). When compared over the four data sets, molecule size does not influence Data Warrior, nSPS, SCS, SAscore or MC1 but affects partly FCFP4, SPS, and more strongly Böttcher and Proudfoot.

**Figure 2:**
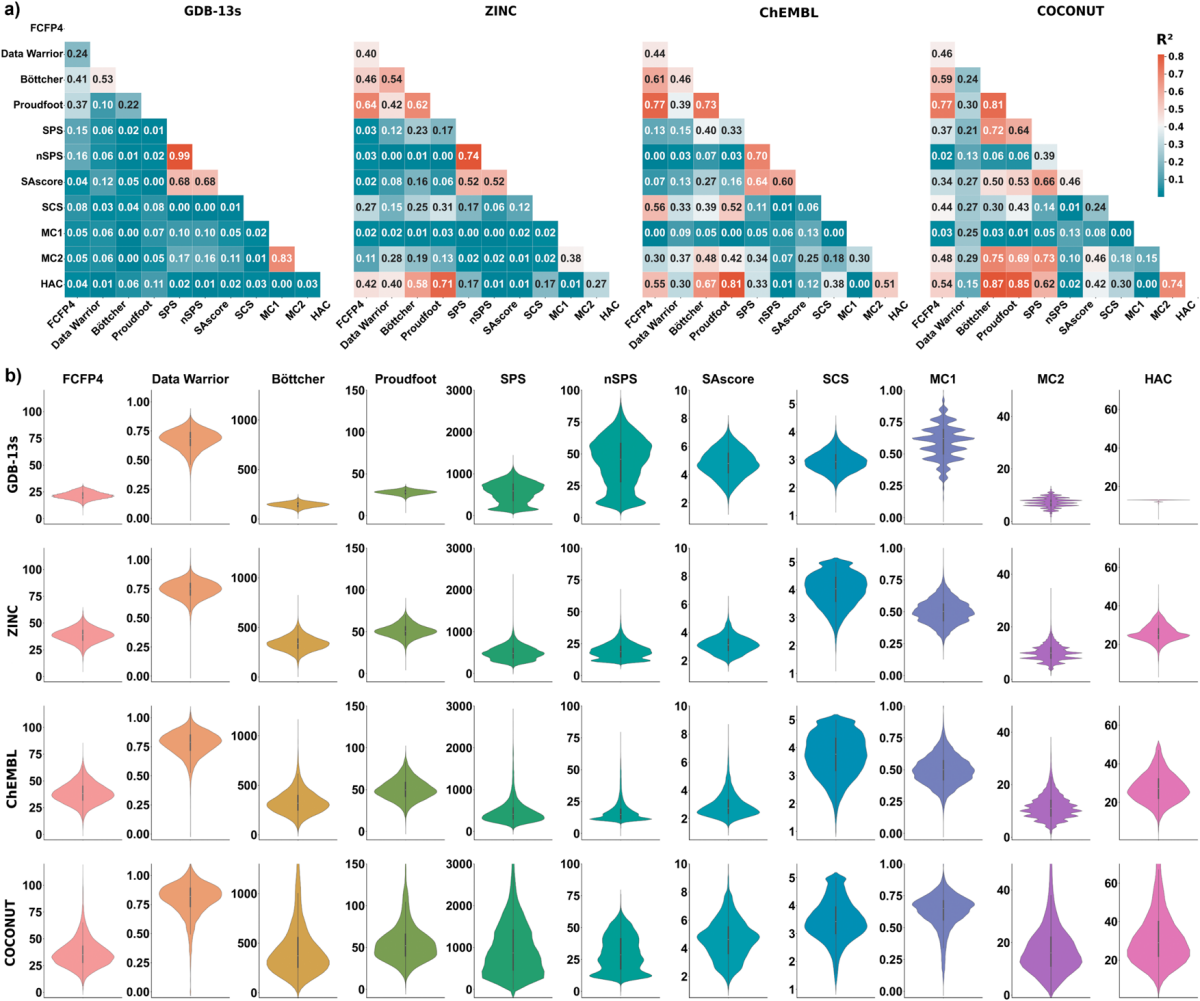
Comparison of MC1 and MC2 with other molecular complexity measures, synthetic accessibility scores, and molecule size. (a) Correlation coefficient matrix between pairs of measures for each data set. The correlation coefficient (*r* ^2^) between pairs of measures is indicated and color-coded (scale at right). (b) Violin plots illustrating the probability density of the data at different values of the complexity or synthesizability measure for each data set. The *y*-axes indicate the values of each respective measure.

The above analysis shows that molecular complexity can be quantified in different ways but that molecule size is an undisputed contributor, which makes sense if complexity relates to synthetic accessibility. The final question remains whether molecular complexity is good or bad. From the point of view of exploring the chemical space for new drugs, we believe that it can be advantageous to start with simple molecules that can be synthesized relatively easily in the laboratory for experimental evaluation, leaving molecular complexity to natural products and drug optimization programs as sources of synthetic challenges. Concerning the practical use of MC1 and MC2, we are currently using them to select GDB molecules for synthetic medicinal chemistry projects and hope that they will find further uses to assist molecule selection in various programs.

## Data and Software Availability

The code for MC1 and MC2 is available at https://github.com/Ye-Buehler/Molecular_Complexity.
